# Supplementary material for: Knowledge, attitude and practice (KAP) and risk factors on dengue fever among children in Brazil, Fortaleza: A cross-sectional study
Source: PLoS Negl Trop Dis. 2023 Sep 25;17(9):e0011110. doi: 10.1371/journal.pntd.0011110 (PMC10553826; doi:10.1371/journal.pntd.0011110)
Supplement: S1 Appendix — (DOCX) [file pntd.0011110.s001.docx]

**S1 Appendix. KAP Survey Questionnaire and Scoring**

| **KAP Domain** | **Question** | **Scoring Method** |
| --- | --- | --- |
| 1.Knowledge | In your opinion, how is dengue spread? | 1 point max  1 if by mosquito; 0.5 subtracted if answered another (incorrect) means of transmission; 0 otherwise |
| 2. Knowledge | Mosquitoes are responsible for dengue transmission. What do you consider to be the main mosquito breeding or egg-laying sites in this community? | 2 points max  2 if 2+ correct answers given; 1 if 1 correct answer given; 0 otherwise |
| 3. Knowledge | Could you tell me what people should do to prevent dengue? | 3 points max  3 if 3+ correct answers given; 2 if 2 correct answers given; 1 if 1 correct answer given; 0 otherwise |
| 4. Knowledge | What do you think are the main signs and symptoms of dengue? | 3 points max  3 if fever, nausea, aches/pains, and rash given; 2 if fever plus at least one of nausea, aches/pains, and rash given; 1 if fever given but not nausea, aches/pains, and rash; 0 otherwise |
| 5. Knowledge | On the last visit from the entomological/dengue agent, were you given any instructions on how to prevent mosquitoes? | 1 if yes, 0 otherwise |
| 6. Attitude | In your opinion, are there ways that dengue can be prevented? | 1 point max  1 if yes; 0 otherwise |
| 7. Attitude | In your opinion, do you think that dengue is a problem for you and your household? | 2 points max  5-choice Likert scale: 2 if strongly agree, 0  if strongly disagree with gradations of 0.5 |
| 8. Attitude | How much danger do you feel that you have of getting sick from dengue? | 2 points max  5-choice Likert scale: 2  if all of the time, 0 if none of the time with gradations of 0.5 |
| 9. Attitude | Has an entomological/dengue agent with the machine on the back ever applied pesticide or “smoke” inside your home since January 2019? | 1 if yes, 0 otherwise |
| 10. Attitude | Do you think that this community can prevent dengue on its own? | 2 points max  5-choice Likert scale: 2 if strongly agree, 0  if strongly disagree with gradations of 0.5 |
| 11. Practice | If you or someone in your family suspects dengue, what do you do (do you see someone, do you take anything or do anything to improve)? | 2 points max  Sum of the following, divided by 3: 3 if seek medical care at health center, hospital, or doctor’s office; 1 if take headache/fever medicine; 1 if increase liquid intake; 1 if go to pharm |
| 12. Practice | What are your and your family’s main sources of information about mosquito control and dengue? | 2 points max  1 for each following answers: Doctor(nurse), school, Entomological/dengue agent, Health agent, Health center  0.5 for each following answers: community leader, friends, family, newspapers, television, radio, internet  0 otherwise(refused, does not know, religious leader) |
| 13. Practice | In this house, in the last 30 days, what did you/your household do to reduce the presence of mosquitoes? | 2 points max  Sum of the following, divided by 3: number of actions taken to control larvae stage (0 if 0; 1 if 1, 2 if 2; 3 if 3+); the adult stage (0 if 0; 1 if 1; 2 if 2+); 1 if shared knowledge with others |
| 14. Practice | When was the last time a larvicide (powder put in water tank and/or container) was used in your house? | 2 points max  2 if within 30 days of taking survey, 1 if more than 30 days prior to taking survey; 0 if no date recalled or never |
| 15. Practice | If larvicides were used, who usually puts it in the containers? | 1.5 points max  1.5 if both; 1 if someone in the household; 0.5 if an entomological/dengue agent; 0 if N/A or does not know. |
| 16. Practice | Has the pesticide or “smoke” car passed on this street where you live since Jan 2019? | 1 if yes, 0 otherwise |
