# Supplementary material for: Knowledge, attitude and practice (KAP) and risk factors on dengue fever among children in Brazil, Fortaleza: A cross-sectional study
Source: PLoS Negl Trop Dis. 2023 Sep 25;17(9):e0011110. doi: 10.1371/journal.pntd.0011110 (PMC10553826; doi:10.1371/journal.pntd.0011110)
Supplement: S2 Appendix — (DOCX) [file pntd.0011110.s002.docx]

**S2 Appendix. Household and Children’s Sample Size per Borough**

| **Children** | **Total**  **N (%)** |
| --- | --- |
| **Neighbourhood** |  |
| Serrinha | 127 (26.3%) |
| Henrique Jorge | 99 (20.5%) |
| João XXIII | 97 (20.1%) |
| Demócrito Rocha | 88 (18.2%) |
| Quintino Cunha | 24 (5.0%) |
| Bonsucesso | 18 (3.7%) |
| Parangaba | 12 (2.5%) |
| Vila União | 9 (1.9%) |
| Damas | 6 (1.2%) |
| Rodolfo Teófilo | 3 (0.6%) |
| **Households** | **Total (N=392)** |
| **Neighbourhood** |  |
| Serrinha | 106 (27.0%) |
| Henrique Jorge | 78 (19.9%) |
| João XXIII | 75 (19.1%) |
| Demócrito Rocha | 73 (18.6%) |
| Quintino Cunha | 22 (5.6%) |
| Bonsucesso | 14 (3.6%) |
| Parangaba | 10 (2.6%) |
| Vila União | 7 (1.8%) |
| Damas | 5 (1.3%) |
| Rodolfo Teófilo | 2 (0.5%) |
