# Supplementary material for: Knowledge, attitude and practice (KAP) and risk factors on dengue fever among children in Brazil, Fortaleza: A cross-sectional study
Source: PLoS Negl Trop Dis. 2023 Sep 25;17(9):e0011110. doi: 10.1371/journal.pntd.0011110 (PMC10553826; doi:10.1371/journal.pntd.0011110)
Supplement: S3 Appendix — (DOCX) [file pntd.0011110.s003.docx]

**S3 Appendix. Sociodemographic Characteristics of Children and Households After Imputation**

| **Children** | **Total (N=483)** |
| --- | --- |
| **Borough** |  |
| Serrinha | 127 (26.3%) |
| Henrique Jorge | 99 (20.5%) |
| João XXIII | 97 (20.1%) |
| Demócrito Rocha | 88 (18.2%) |
| Quintino Cunha | 24 (5.0%) |
| Bonsucesso | 18 (3.7%) |
| Parangaba | 12 (2.5%) |
| Vila União | 9 (1.9%) |
| Damas | 6 (1.2%) |
| Rodolfo Teófilo | 3 (0.6%) |
| **Child Age, year** |  |
| Mean (SD)  Median [Min, Max] | 6.54 (2.74)  6.00 [2.00, 12.0] |
| 2-5 | 181 (50%) |
| 6-12 | 181 (50%) |
| **Child Race** |  |
| Multiracial | 313 (64.8%) |
| White | 127 (26.3%) |
| Black | 31 (6.4%) |
| Yellow | 12 (2.5%) |
| **Child Gender** |  |
| Female | 222 (46.0%) |
| Male | 261 (54.0%) |
| **School Attendance** |  |
| Yes | 431 (89.2%) |
| No | 50 (10.4%) |
| Refused | 2 (0.4%) |
| **Households** | **Total (N=392)** |
| **Borough** |  |
| Serrinha | 106 (27.0%) |
| Henrique Jorge | 78 (19.9%) |
| João XXIII | 75 (19.1%) |
| Demócrito Rocha | 73 (18.6%) |
| Quintino Cunha | 22 (5.6%) |
| Bonsucesso | 14 (3.6%) |
| Parangaba | 10 (2.6%) |
| Vila União | 7 (1.8%) |
| Damas | 5 (1.3%) |
| Rodolfo Teófilo | 2 (0.5%) |
| **Household Monthly Income (BRL)*** |  |
| Low | 22 (5.6%) |
| Moderate | 283 (72.2%) |
| High | 87 (22.2%) |
| **Respondent Education** |  |
| Lower than Elementary School | 116 (29.6%) |
| Elementary School or Higher | 276 (70.4%) |
| **Respondent Gender** |  |
| Female | 348 (88.8%) |
| Male | 44 (11.2%) |
| **Respondent Race** |  |
| Black | 38 (9.7%) |
| Multiracial | 287 (73.2%) |
| White | 56 (14.3%) |
| Asian | 10 (2.6%) |
| Indigenous | 1 (0.3%) |
| **Occupation** |  |
| Retired/Unemployed/Other | 202 (51.5%) |
| Employed | 190 (48.5%) |
| **Household KAP** |  |
| Low | 101 (25.8%) |
| Moderate | 271 (69.1%) |
| High | 20 (5.1%) |
| *BRL= Brazilian real |  |
