# Supplementary material for: Knowledge, attitude and practice (KAP) and risk factors on dengue fever among children in Brazil, Fortaleza: A cross-sectional study
Source: PLoS Negl Trop Dis. 2023 Sep 25;17(9):e0011110. doi: 10.1371/journal.pntd.0011110 (PMC10553826; doi:10.1371/journal.pntd.0011110)
Supplement: S4 Appendix — (DOCX) [file pntd.0011110.s004.docx]

**S4 Appendix. Mean Percentage Score of KAP Questions with 95% Confidence Interval**

| **Knowledge Domain Question** | **Mean Percentage Score (95% CI) *** |
| --- | --- |
| In your opinion, how is dengue spread? | 83% (69% - 97%) |
| Mosquitoes are responsible for dengue transmission. What do you consider to be the main mosquito breeding or egg-laying sites in this community? | 68% (54% - 82%) |
| Could you tell me what people should do to prevent dengue? | 68% (54% - 82%) |
| What do you think are the main signs and symptoms of dengue? | 84% (70% - 98%) |
| On the last visit from the entomological/dengue agent, were you given any instructions on how to prevent mosquitoes? | 68% (54% - 82%) |
| Overall | 74% (61% - 88%) |
| **Attitude Domain Question** |  |
| In your opinion, are there ways that dengue can be prevented? | 98% (84% - 100%) |
| In your opinion, do you think that dengue is a problem for you and your household? | 86% (72% - 100%) |
| How much danger do you feel that you have of getting sick from dengue? | 57% (44% - 71%) |
| Has an entomological/dengue agent with the machine on the back ever applied pesticide or “smoke” inside your home since January 2019? | 14% (0%- 28%) |
| Do you think that this community can prevent dengue on its own? | 41% (37% - 45%) |
| Overall | 63% (46% - 74%) |
| **Practice Domain Question** |  |
| If you or someone in your family suspects dengue, what do you do (do you see someone, do you take anything or do anything to improve)? | 50% (36% - 64%) |
| What are your and your family’s main sources of information about mosquito control and dengue? | 67% (53% - 81%) |
| In this house, in the last 30 days, what did you/your household do to reduce the presence of mosquitoes? | 25% (11% - 39%) |
| When was the last time a larvicide (powder put in water tank and/or container) was used in your house? | 19% (17% - 22%) |
| If larvicides were used, who usually puts it in the containers? | 19% (17% - 20%) |
| Has the pesticide or “smoke” car passed on this street where you live since Jan 2019? | 60% (46% - 74%) |
| Overall | 39% (25% - 53%) |

* By dividing the score of each household achieved by the maximum points of each question
