# Supplementary material for: Knowledge, attitude and practice (KAP) and risk factors on dengue fever among children in Brazil, Fortaleza: A cross-sectional study
Source: PLoS Negl Trop Dis. 2023 Sep 25;17(9):e0011110. doi: 10.1371/journal.pntd.0011110 (PMC10553826; doi:10.1371/journal.pntd.0011110)
Supplement: Attachment — Submitted filename: Dengue_Response.docx [file pntd.0011110.s005.docx]

Dear Dr. Wang,

We appreciate the opportunity to revise our manuscript and also appreciate the thoughtful comments provided by the reviewers, which have enhanced the quality and clarity of our manuscript. We have carefully considered each comment and we hope that our responses and changes have adequately addressed your concerns as well as the reviewers’ concerns.

Kate Zinszer PhD, MSc

Associate Professor

School of Public Health

University of Montreal

kate.zinszer@umontreal.ca

**Reviewer #1**

**1. The sample size for serology is rather small (<500 children) and I note that in the methods further down the intention was to do ten times more clusters and people. Whilst we all appreciate the pandemic problems, it unfortunately highlights the problem of the sample size. One would presume that some power calculations had been done for the larger wished for sample size, but the study is now clearly under-powered. It is also unclear whether the study that aimed for a 10 times greater sample size is being pursued or that simply these were the only samples that could be obtained during the project time frame. Could the authors perhaps do a retrospective power calculation based on their initial power calculation to reveal what power the small achieved number of samples could be expected to achieve?**

Thank you for the comment and we appreciate your concern about the sample size. The 10x larger sample size was calculated for the primary objective of the cluster randomized trial (cRCT) and prior to data collection. For the primary objective of the cRCT, power calculations were based on the minimum clinically meaningful difference in prevalence of 0.2115 in those with the intervention and 0.30 in those without the intervention. The study in Fortaleza is terminated, so data collection has ceased, and we are restricted to these (partial) baseline data.

The current KAP study objective is part of a secondary objective. In this post-hoc analysis, we are comparing seroprevalence between low, medium and high KAP score categories, we are using the data available, and we are basing our understanding of any power limitations on the standard errors and width of the confidence intervals.

Nevertheless, we have performed retrospective power calculations based on the seroprevalences observed for each KAP category and, separately on the minimum clinically meaningful prevalence stated in the original intervention study protocol i.e. 0.2115 vs 0.3.

Calculation:

The power calculation inputs are the seroprevalence of each KAP category pair, the ICC, the coefficient of variation for the cluster sizes, and the average cluster sample size.

Seroprevalences by KAP category are 0.21, 0.26 and 0.39 for the low, medium and high KAP categories, respectively.

We have an ICC (Lohr rho) of 0.03

The coefficient of variation is (SD of cluster sizes divided by the mean), 37.7/40.25 = 0.94

With 12 clusters, a coefficient of variation of 0.94, a significance level of 0.05, and an average of 40 subjects per cluster, we would have 91 % power to detect a difference between the High and Low KAP group seroprevalences of 0.39 and 0.21, respectively. We have only 16% power to detect a difference of 0.26 and 0.21 prevalence between the medium and low KAP groups.

The original cluster randomized clinical trial considered differences based on seroprevalences of 0.21 (intervention group) and 0.30 (control group). The power to detect this difference is only 39%. However, as mentioned, this is not the objective of the current research study.

We modified the text in the manuscript to clarify(Page 4 Method Section):

“The cRCT was powered for the primary objective of comparing seroprevalence between those receiving the intervention and those not receiving the intervention.19 The current study is part of a secondary objective, therefore a power analysis was not conducted. The cRCT planned to collect data from 68 clusters with a target recruitment of 5,848 children between 2 and 12 years old. For further detail, please see the original trial protocol in Zinszer et. al. 202019. Due to the Covid-19 pandemic and instability in the study region, the baseline data collection terminated before data from all clusters were gathered. The team was able to collect complete data from four clusters and partial data from eight clusters (from 10 different neighbourhoods in Fortaleza), between November 2019 and February 2020.”

**2. It would be useful to indicate the age of the children in parentheses (it would seem from further down that children assessed for serology are 2-12 years old). Why this age group and not for example up to 18 years of age. Are** **these groups based on previous seroprevalence data? And which thus provide the basis for the sample size calculations?**

Yes, these groups based on previous seroprevalence data. A preliminary analysis of a pilot data in Fortaleza (conducted by one of the co-PIs, Antonio S. Lima Neto) showed that 35% of children from 3 to 9 years old were positive for anti-dengue IgG detection at baseline, which increased to 46% in 10 years old (with an average 52% positivity among 10 to 15-year-olds). Therefore, initially, the range from 3 to 9 years old was chosen to have the largest percentage of seronegative children to follow through the study period.

After the start of data collection, we verified that, due to the demographic transition in the region since the pilot data collection, our sample size objectives per census sector would not be reached if only children from 3 to 9 years old were included. Then, in December 2019, an amendment to increase the age range to 2 to 12 years old to improve recruitment was submitted and approved by both IRBs (UECE and UDEM).

**3. As mentioned above, this was meant to be a larger study and part of a cluster randomized control trial published by the authors in Trials. Can the authors explain how a cluster was defined, insofar as here they achieved sampling from 4 clusters.**

In Fortaleza, there are 3,020 census tracts with approximately 230 households and 810 individuals in each census tract. Each cluster was enumerated and complied with eligibility criteria. The selection criteria for clusters included:

1) Any of the 3,020 census tracts from the 2010 Census and EXCLUDES:

2) Census tracts where interventions outside of vector control standard practices occurred within the last 5 years;

3) Census tracts deemed to be too insecure for study personnel (determined based upon the opinion of the research team and stakeholders)

4) Clusters with less than 120 households as previous work has shown that 120 households are required to obtain the needed sample size.

Then, clusters were selected through a proportionate stratified random sampling based on a dengue risk index calculated from the cumulative incidence of dengue notifications from 2016 to 2018 per neighbourhood or bairro (census tracts within each bairro were assigned to the same risk strata).

There were 68 total clusters or census tracts with 12 clusters being included in this analysis (4 fully and 8 partially collected clusters, from 10 neighbourhoods throughout Fortaleza) due to instability in the study country and the emergence of the Covid-19 pandemic. All households in selected clusters with children from 2 to 12 years old were considered eligible and asked to participate in the trial. Detailed inclusion and exclusion criteria can be found in the study protocol^1^.

Please see the following text in the manuscript (Page 4 Method section):

“There are 3,020 census tracts (clusters) in Fortaleza and each cluster was enumerated and filtered by eligibility criteria. Eligible clusters were then categorized according to the dengue vulnerability index (DVI), based on the 2010 Census. To limit contamination among clusters, any selected cluster with common boundaries to previously selected clusters was excluded and replaced by the next cluster (from a ranked list based upon stratification criteria).”

**4. I fail to see an ethics statement about written and oral (?) informed consent for parents/guardians and their children, although I see further down the ethical approval by the pertinent ethics committees.**

Thank you for the comment, the following text has been added to the manuscript (Page 4,5 Eligibility Criteria and Study Procedure):

“There were two different informed consent forms (ICF) and one assent form. The first ICF was signed (or digit printed if not able to write) by the person responsible for the household and able to answer the household questionnaire (principal adult respondent). For each participating child, a specific ICF for serology blood collection was signed by the child’s parent or guardian. Study objectives and the details of the procedures were explained in adapted language by trained study staff and an assent form was signed or fingerprinted by the participating child if they agreed to participate. Households and children with contaminated blood samples and/or with indeterminate serology results were excluded from this analysis.”

**5. The justification behind the methodology and scoring of the KAP study is not clearly defined and the reference given from a Saudi Covid study does not seem to be adequate as a reference methodology. There are many KAP methodology papers that can be referred to. A recent paper with very many pertinent references can be found here:** [**https://can01.safelinks.protection.outlook.com/?url=https%3A%2F%2Fdoi.org%2F10.1016%2Fj.envres.2020.110509&data=05%7C01%7Cyang.zhang8%40mail.mcgill.ca%7Cfba37a93b5704cff4dda08db29d2ef7c%7Ccd31967152e74a68afa9fcf8f89f09ea%7C0%7C0%7C638149758157716813%7CUnknown%7CTWFpbGZsb3d8eyJWIjoiMC4wLjAwMDAiLCJQIjoiV2luMzIiLCJBTiI6Ik1haWwiLCJXVCI6Mn0%3D%7C3000%7C%7C%7C&sdata=a1O3Ve8vUtRq0R25OdVj4wK%2BOBLR88PHR3zhO9sFYqE%3D&reserved=0**](https://can01.safelinks.protection.outlook.com/?url=https%3A%2F%2Fdoi.org%2F10.1016%2Fj.envres.2020.110509&data=05%7C01%7Cyang.zhang8%40mail.mcgill.ca%7Cfba37a93b5704cff4dda08db29d2ef7c%7Ccd31967152e74a68afa9fcf8f89f09ea%7C0%7C0%7C638149758157716813%7CUnknown%7CTWFpbGZsb3d8eyJWIjoiMC4wLjAwMDAiLCJQIjoiV2luMzIiLCJBTiI6Ik1haWwiLCJXVCI6Mn0%3D%7C3000%7C%7C%7C&sdata=a1O3Ve8vUtRq0R25OdVj4wK%2BOBLR88PHR3zhO9sFYqE%3D&reserved=0)

Thank you for the comment. Although KAP surveys are frequently used for dengue studies, there is no standard approach to analyzing the data, and cutoffs are usually set arbitrarily by researchers. Given the lack of best practice (or consolidated guidance) in selecting cutoffs, we adopted cut-off points from a COVID KAP study (≤ 50%, 50% - 75%, and >75%) which we believed would provide more information than using a single cut-off point, e.g., in Rahman et al.,(2020)^2^.

We agree that we did not cite enough references for the methodology and scoring of the KAP, and we appreciate the paper you have recommended. More details have been added to the “Knowledge, Attitudes, and Practice” section under Methods and multiple new KAP dengue studies with the same or similar cut-offs have been cited(Page 5 Knowledge, Attitude and Practice)^3,4^.

**6. As mentioned above, there lacks a sample size calculation.**

Please refer to the answer for the first comment.

The following text was added to the manuscript.

“The cRCT was powered for the primary objective of comparing seroprevalence between those receiving the intervention and those not receiving the intervention. The current study is part of a secondary objective, so a power analysis was not carried out. The cRCT planned to collect data from 68 clusters with a target recruitment of 5,848 children between 2 and 12 years old. For further detail, please see the original trial protocol in Zinszer et. al. 2020. Due to the Covid-19 pandemic and instability in the study region, the baseline data collection terminated before data from all clusters were gathered. The team was able to collect complete data from four clusters and partial data from eight clusters (from 10 neighbourhoods throughout Fortaleza), between November 2019 and February 2020.”

**7. Elution of the immunoglobulins from the bloodspot for the PanBio ELISA. Can the authors write how they eluted the IgGs from the dried blood spot?**

More details have been added to the section of “seroprevalence test” under Methods (Page 6).

“At the laboratory, three punches of 6 mm from the DBS were soaked in a 1.5 mL vial containing 250 µL of eluent solution, shaken overnight at 4 °C to ensure adequate serum elution. The next day, the eluates were centrifuged at 2,500 rpm for 2 minutes in a refrigerated microfuge and then transferred to a clean and sterile tube. Undiluted DBS eluates were treated as a 1:25 equivalent dilution of human serum, based on previous work that demonstrated that approximately three 6 mm circles contained 21 µL of dried blood, corresponding to approximately 10.5 µL of serum that were eluted in 250 µL of the eluent solution.”

**8. More information on the KAP questions: there are very few and so perhaps these can be spelled out and justified.**

The KAP questionnaires have been added to the supplementary information, and a more detailed explanation on how scores were assigned has been added to the KAP section under Method (Page 5).

“For the multiple choice and dichotomous questions, only correct responses were assigned positive scores (an incorrect response was given a score of zero). For Likert scale questions, the highest score was assigned for ‘strongly agreed’, 0 for ‘strongly disagree’ with gradations of 0.5 for 5-choice Likert scale questions.”

The study questionnaires were designed to capture multidisciplinary information with questions about sociodemographics, recent dengue episodes, KAP, social capital, acceptability and participation in research activities, to determine if interventions based upon community mobilization reduce the risk of dengue virus infection among children. Thus, there were only 16 questions for KAP. However, we believe this is not a weakness of our study as there have been existing studies with a similar number of KAP questions^2,4,5^, and some of our KAP questions capture more information with a maximum score of more than 1(2 or 3) instead of a simple Yes/No question, which allows us to obtain adequate information to assess the participating households’ KAP.

**9. Statistical analysis: as one might expect KAP variables to be somewhat correlated, were there any aeempts to look at co-linearity and perhaps use of Principle components analyses to generate novel variables that are comprised of like KAP variables?**

Regarding multicollinearity and a principal components analysis, the KAP variables were already combined as a score, based on a method described in a paper cited in our manuscript, and the score was categorized as low, medium, and high. Multicollinearity was assessed for the final model and was found to be low. Though the idea of finding a new score for KAP based on principal components is interesting, we followed the general approach used in other studies. We also hoped to compare our results to those of the other study, in order to capture what is lacking among the study population in terms of KAP to provide insight for future interventions.

**10. As there were more children than households, there must have been multiple children from a single household. How was this taken into account in the analyses?**

Eighty-seven percent of households contributed one child to the study. We initially used a quasi-binomial model to account for clustering/overdispersion but the dispersion parameter was 1.0, providing no evidence of overdispersion from clustering. With such a small percentage of households with more than one child and little evidence of overdispersion, accounting for household clustering would be inefficient and would make it difficult to meet the methodological standard of estimating the relative risk directly (rather than using a logistic regression), when the outcome (seropositivity) is common. Please see the response to Reviewer 1 in the Results section for further detail.

**Reviewer #3:**

**1.The aim and objectives are clear. The methods are well written and largely reproducible. The authors acknowledge that the sample size was not met due to the pandemic (would probably specify Covid-19) and instability in the region. Suggest to specify that full details on the methods / study population can be found in Zinszer et al., (2020)**

Thank you for the comment, more details regarding methods/study population have been added to the manuscript. Please note that the methods in the 2020 publication^1^ are focused on the primary objective of the intervention trial and the methods in the current study are for a post hoc analysis.

We added the following text to the manuscript:

“For further detail, please see the original trial protocol in Zinszer et. al^1^. Due to the Covid-19 pandemic and instability in the study region, the baseline data collection terminated before data from all clusters were gathered.”

**2. Also to specify how a cluster is defined and selected. Further, it would be useful to know whether the clusters included were randomly distributed throughout the city (or concentrated in a specific area etc.).** 

Please refer to the response to Reviewer#1, comment#3 for cluster selection, more details regarding the clusters included for the analysis have been added to the manuscript. The sample size per borough were added to Table 1.

The clusters included were randomly distributed throughout the city, below is a table summary of clusters (boroughs) included for the analysis.

The following text was added to the manuscript:

“There are 3,020 census tracts (clusters) in Fortaleza and each cluster were enumerated and filtered by eligibility criteria. Eligible clusters were then categorized according to the dengue vulnerability index (DVI), based on the 2010 Census. To limit contamination among clusters, any selected cluster with common boundaries to previously selected clusters was excluded and replaced by the next cluster (from a ranked list based upon stratification criteria).”

**3. It would also be useful for other researchers to include the KAP questionnaire in the supplementary information. The sample size calculation (given the reduction in sample size is not presented).**

Thank you for the suggestion, the KAP questionnaire is now included in the supplementary information. For the sample size calculation, please refer to the response to Reviewer#1, comment#1.

The following text has been added to the manuscript (Page 4 Method Section):

“The cRCT was powered for the primary objective of comparing seroprevalence between those receiving the intervention and those not receiving the intervention.19 The current study is part of a secondary objective, therefore a power analysis was not conducted. The cRCT planned to collect data from 68 clusters with a target recruitment of 5,848 children between 2 and 12 years old. For further detail, please see the original trial protocol in Zinszer et. al. 202019. Due to the Covid-19 pandemic and instability in the study region, the baseline data collection terminated before data from all clusters were gathered. The team was able to collect complete data from four clusters and partial data from eight clusters (from 10 different neighbourhoods in Fortaleza), between November 2019 and February 2020.”

**Results**

**Reviewer #1:**

**The results are very interesting, especially given the small sample size.
It would be very useful to have more information about the KAP results at a single variable level. Just presenting a KAP score is too brief.**

Thank you for the comment. More information and tables about the KAP results have been added to the result section.

Results section (Page 13):

“For each KAP question, Tables 4-6 display the mean percentage score with 95% confidence intervals. The percentage score was calculated by dividing the score of each household achieved by the maximum points of each question. Out of the three domains, the knowledge domain had the highest mean percentage score of 74%, followed by the attitude domain and then the practice domain at 63% and 39%, respectively. While all questions in the knowledge domain have a mean percentage score over 65%, the mean percentage score varies largely among questions in the attitude and practice domains.”

Discussion Section (Page 19)

Participating households demonstrated a good understanding of vector control and dengue transmission, prevention, and symptoms, as evidenced by the relatively high overall mean percentage score in the knowledge domain compared to the knowledge level observed in previous studies conducted in dengue pandemic areas.^20,59,60^ This phenomenon can likely be attributed to the high prevalence of dengue fever in Brazil and exposure to dengue prevention education campaigns and related information.

In terms of attitudes, a fair overall mean score was observed. Almost all participating respondents believe they were at-risk of dengue and that dengue can be prevented although the majority of participants lacked confidence in the community's dengue prevention capabilities. This attitude could be due to several factors, which include a lack of support from government and community services^61-63^ and disillusionment about household and community agency^64,65^. There was poor level of household-level vector control practices and in certain studies, it was demonstrated that knowledge was not associated with any significant behavior change.^66,70^ Given the low confidence community prevention ability among the study population, community engagement activities may be necessary to deliver key messages on dengue and to work with the community to identify acceptable vector control interventions.^66-68^ This would ensure the sustainability of long-term community dengue prevention interventions.

Table 4. Mean Percentage Score of Knowledge Domain Questions

| **Question** | **Mean Percentage Score (95% CI) *** |
| --- | --- |
| In your opinion, how is dengue spread? | 83% (69% - 97%) |
| Mosquitoes are responsible for dengue transmission. What do you consider to be the main mosquito breeding or egg-laying sites in this community? | 68% (54% - 82%) |
| Could you tell me what people should do to prevent dengue? | 68% (54% - 82%) |
| What do you think are the main signs and symptoms of dengue? | 84% (70% - 98%) |
| On the last visit from the entomological/dengue agent, were you given any instructions on how to prevent mosquitoes? | 68% (54% - 82%) |
| Overall | 74% (61% - 88%) |

* By dividing the score of each household achieved by the maximum points of each question

Table 5. Mean Percentage Score of Attitude Domain Questions

| **Question** | **Mean Percentage Score (95% CI) *** |
| --- | --- |
| In your opinion, are there ways that dengue can be prevented? | 98% (84% - 100%) |
| In your opinion, do you think that dengue is a problem for you and your household? | 86% (72% - 100%) |
| How much danger do you feel that you have of getting sick from dengue? | 57% (44% - 71%) |
| Has an entomological/dengue agent with the machine on the back ever applied pesticide or “smoke” inside your home since January 2019? | 14% (0%- 28%) |
| Do you think that this community can prevent dengue on its own? | 41% (37% - 45%) |
| Overall | 63% (46% - 74%) |

* By dividing the score of each household achieved by the maximum points of each question

Table 6. Mean Percentage Score of Practice Domain Questions

| **Question** | **Mean Percentage Score (95% CI) *** |
| --- | --- |
| If you or someone in your family suspects dengue, what do you do (do you see someone, do you take anything or do anything to improve)? | 50% (36% - 64%) |
| What are your and your family’s main sources of information about mosquito control and dengue? | 67% (53% - 81%) |
| In this house, in the last 30 days, what did you/your household do to reduce the presence of mosquitoes? | 25% (11% - 39%) |
| When was the last time a larvicide (powder put in water tank and/or container) was used in your house? | 19% (17% - 22%) |
| If larvicides were used, who usually puts it in the containers? | 19% (17% - 20%) |
| Has the pesticide or “smoke” car passed on this street where you live since Jan 2019? | 60% (46% - 74%) |
| Overall | 39% (25% - 53%) |

* By dividing the score of each household achieved by the maximum points of each question

**Reviewer #3:**

**1. The results are presented well. Multivariable regression models are more commonly constructed as logistic regression and odds ratios presented. Does the multivariate model account for within-cluster correlation? It might be better to perform logistic regression with random effects for the multivariate risk factor analysis.**

We appreciate your concern regarding within-cluster correlation.

Regarding overdispersion due to clustering, we started by running a quasibinomial model but saw little evidence of overdispersion after accounting for the covariates because the dispersion parameter from the quasibinomial was 1.0, output below.

Quasibinomial output:

There are also only 12 clusters, several with small sample sizes (n= 3, 6, 9, 12, 12, 18, 24, 41, 86, 87, 88, 97) so model non-convergence is an issue^6^. For clustering by household, eighty-seven percent of households had one child in the study and there were 392 households. Binary outcome models are more subject to bias and non-convergence when the group sample size is small particularly when most groups have only one member^6^. Random effects models are not advised when there are small cluster sizes or a small number of clusters.

For these reasons, we have little justification for including random effects for cluster or household. Even if the model algorithms converged and the model appeared stable, this would likely use power with little benefit and may result in biased estimates.

We selected Zou’s modified Poisson regression (rather than logistic regression) because it directly estimates the relative risk and the logistic regression model odds ratios (as estimates of relative risk) are biased away from the null when the outcome is common. This approach has been recommended for binomial outcomes with an overall prevalence (probability of success) over 0.10^7-9^. Our overall seroprevalence is 0.25 so we feel this is an appropriate choice.

In the model results below, when comparing the logistic random effects model odds ratios and the modified Poisson relative risks, we see that several of the logistic regression ORs are biased estimates of relative risk – inflating the effect of the covariate on the outcome. For this reason, we believe our model choice is appropriate.

Logistic output:

Zou’s modified Poisson displaying relative risks (not odds ratios)

**2. It would probably be useful for the reader to have the KAP results added to supplementary information so that they can see which parts scored high / low.**

Thank you for the suggestion, please refer to the response to reviewer#1’s comment for the result section.

**Conclusions**

**Reviewer #1: Likewise the discussion on the KAP findings is almost non-existent. As stated perhaps the respondents have higher KAP scores because they are more confronted by dengue. So dengue occurrence drives KAP. However, whilst history of dengue might improve knowledge, did it improve attitude and practices?**

Thank you for pointing this out, we modified the section discussing how past dengue history might impact KAP as a whole, and added a paragraph to the manuscript discussing the impacts of the history of dengue on attitude and practices.

“An interesting finding from our study is that children living in households with high KAP levels had a higher relative risk of dengue seropositivity. One hypothesis for this finding is related to our outcome measure and an important limitation of cross-sectional data - a lack of temporality. We did not measure active dengue infections but measured dengue seropositivity using IgG, which can persist in the blood for several years. It is plausible that this inverse association is due to a mismatch of temporality between our outcome and exposure of interest.

Cohort and case-control studies have found participants who had experience with dengue had a significantly higher knowledge score, as they are likely to seek for information when encountering the disease or have more chances to learn from healthcare staff when receiving treatments. Among children with dengue experiences, they are more likely to perceive dengue as a serious concern. Studies conducted in Indonesia and Colombia have shown that adults who have experienced dengue are also more likely to have a positive attitude toward preventing it. In terms of the impact of dengue experience on prevention practices, a study from Malaysia found age and dengue history are the main determinants that influence the high practice level, which is consistent with another study’s result where dengue experience was found to be statistically positively associated with dengue prevention practices. Therefore, if a child had a previous dengue infection, it is likely that this experience improved the overall KAP in the household.”

**Reviewer #3: The discussion is well written and acknowledges study limitations. It would be good to discuss the impact of the pandemic / reduction in target numbers again in the study limitations. The discussion focuses on seropositivity and associations with KAP, it would also be useful to include some general results/recommendations regarding KAPs and the study population. So were the KAP scores in the study population high/low – was there particularly areas which had low awareness which could be targeted for improvement.**

Thank you for the suggestion, please refer to the response to reviewer#1’s comment for the result section.

**Summary and General Comments**

**Reviewer #1: An interesting pilot study on a subject of interest, namely relating KAP to dengue seropositivity. However, through clearly collateral covid-associated damage, the sample sizes achieved are too small to be very meaningful. As a pilot study this is OK.**

**1. This is a study relating KAP, socioeconomics and dengue seropositivity. A recent article in PNTD in India looked exactly at this question and including the precise aspects on access to drinking water stated in the present study. I would suggest the authors read this article and the references cited within to put their study into what has been recently published on this: doi: 10.1371/journal.pntd.0009024.**

Thank you for your suggestion. We have read the article and added references accordingly.

For the comment regarding its adequacy as a pilot study, as answered above, we believe the sample size for this analysis is adequate, especially considering the research objective is not to evaluate the effectiveness of an intervention as originally planned in the study protocol, but to estimate the dengue seropositivity for study participants and also to examine the association between households’ dengue-related KAP with children’s risk of dengue seropositivity while accounting for socioeconomic and demographic differences. We acknowledge that further investigation is needed to examine other associations that we were not able to capture, for example, factors that influence poor preventive practices and barriers to community interventions implementation.

**Abstract**

**2. Abstract line 2 : It reads « Children are vulnerable to infection with the dengue virus and the risk of severe dengue disease is the highest among infants and children, particularly those who have been previously infected with a different dengue serotype.”**

**This isn’t actually true and it depends very much on the force of infection. Even in many endemic countries the average age of first infection can be as late as 10 years old and the increased disease severity following a secondary infection thus occurs later into young adulthood. The key point is that age-severity is very much context dependent.**

Thank you so much for pointing this out, changes have been made accordingly.
“Children are vulnerable to infection with the dengue virus, particularly those who have been previously infected with a different dengue serotype.”

**3. The section introducing KAP is a little limited and lacks references with respect to dengue. The authors write that there have been several studies on KAP and dengue, not one is cited.**

Thank you for your suggestion. We have incorporated additional references to the content.

**Reviewer #2: In this manuscript, Zhang and colleagues performed a cross-sectional study of dengue seroprevalence in Fortaleza, Brazil between November 2019 and February 2020. They examined the association between characteristics of certain study populations and risk of dengue seropositivity. The authors found that children’s age was most strongly associated with the risk of dengue seropositivity. I have a few suggestions:

(1) It’s not very clear why the authors collected these data for only a single dengue season in the study location. The seasonality of dengue outbreaks can be driven by many factors, including climate conditions and pre-existing population immunity. The authors may wish to examine the influence of these factors on the observed seroprevalence.**

Thank you for the suggestion, we were not able to capture the pre-existing population immunity as the baseline collected was terminated unexpectedly (political instability + Covid-19 pandemic) and therefore the study became cross-sectional. We added a rainy season indicator to account for climate conditions to the regression, based on your suggestion. As the blood sample and responses to the household survey were captured between November 2019 and February 2020, there was a substantial change in the number of rainy days between the end of 2019 and early 2020. We created a new variable “Rain Season” which divided the study population into two groups by the date they were interviewed and when the children’s blood was taken (they were carried out on the same date). The text and tables in the manuscript have been modified accordingly.

**(2) The authors may also wish to examine the difference in seroprevalence between the study period and the same period in previous years.**

Thank you for the comment, unfortunately, we are not able to compare the difference in seroprevalence due to the cross-sectional study design and with IgG seropositive as the primary outcome. Nonetheless, we did compare the seroprevalence among the children who participated with previous studies in the discussion section.

References

1. Zinszer K, Caprara A, Lima A, Degroote S, Zahreddine M, Abreu K, et al. Sustainable, healthy cities: Protocol of a mixed methods evaluation of a cluster randomized controlled trial for Aedes control in Brazil using a community mobilization approach. Trials. 2020;21(1).
2. Khan W, Rahman A, Zaman S, Kabir M, Khan R, Ali W, et al. Knowledge, attitude and practices regarding dengue and its vector among medical practitioners in Malakand Region, Pakistan. Brazilian Journal of Biology. 2023;83.
3. Saghir MA, Ahmed WA, Dhaiban MM, Osman ME, Abduljabbar NI. Knowledge, attitude, and practices of the community toward Dengue fever in Shabwah Governorate, Yemen: A descriptive study. Journal of the Egyptian Public Health Association. 2022;97(1).
4. Jayawickreme KP, Jayaweera DK, Weerasinghe S, Warapitiya D, Subasinghe S. A study on knowledge, attitudes and practices regarding dengue fever, its prevention and management among dengue patients presenting to a tertiary care hospital in Sri Lanka. BMC Infectious Diseases. 2021;21(1).
5. Sarmiento-Senior D, Matiz MI, Jaramillo-Gómez JF, Olano VA, Vargas SL, Alexander N, et al. Knowledge, attitudes, and practices about dengue among pupils from rural schools in an endemic area in Colombia. Biomédica. 2019;39(3):478–90.
6. Moineddin R, Matheson FI, Glazier RH. A simulation study of sample size for multilevel logistic regression models. BMC Medical Research Methodology. 2007;7(1). doi:10.1186/1471-2288-7-34
7. Gallis JA, Turner EL. Relative measures of association for binary outcomes: Challenges and recommendations for the Global Health Researcher. Annals of Global Health. 2019;85(1). doi:10.5334/aogh.2581
8. Chen W, Qian L, Shi J, Franklin M. Comparing performance between log-binomial and robust Poisson regression models for estimating risk ratios under Model misspecification. BMC Medical Research Methodology. 2018;18(1). doi:10.1186/s12874-018-0519-5
9. Yelland LN, Salter AB, Ryan P. Performance of the modified Poisson regression approach for estimating relative risks from clustered prospective data. American Journal of Epidemiology. 2011;174(8):984–92. doi:10.1093/aje/kwr183
