# Supplementary material for: Knowledge, attitude and practice (KAP) and risk factors on dengue fever among children in Brazil, Fortaleza: A cross-sectional study
Source: PLoS Negl Trop Dis. 2023 Sep 25;17(9):e0011110. doi: 10.1371/journal.pntd.0011110 (PMC10553826; doi:10.1371/journal.pntd.0011110)
Supplement: Attachment — Submitted filename: DengueResponse_SecondRound.docx [file pntd.0011110.s006.docx]

Dear Dr. Wang,

Thank you for giving us the opportunity to revise our manuscript and we would also like to thank the reviewers for their very helpful feedback. We have addressed each comment and hope that the manuscript is now acceptable for publication.

Kate Zinszer PhD, MSc

Associate Professor

School of Public Health

University of Montreal

kate.zinszer@umontreal.ca

**Reviewer #2 (No response)**

**Reviewer #3:**

1. **A map of the distribution of the clusters (especially if dengue is influenced by environmental conditions would be useful).**

Thank you for the comment and please refer to the map of the distribution of the 68 clusters below for the CRT and then the lower map shows the clusters that were included in this cross-sectional study. We used a dengue risk index provided at the census tract level to identify potential clusters that were moderate to high risk of dengue infection, and the risk index was provided by the Fortaleza Municipal Health Secretariat. The risk index is based on a mixture of information including historical cases, population density, and poverty and deprivation level. We also included a rainy season indicator to account for differences (in terms of timing of sample collection). We do not anticipate that there would be much temperature and/or rainfall differences between the clusters given their close proximity.

2. **If the within-cluster correlation is not controlled for – I would add the justification for this somewhere in the manuscript.**

Given the low number of clusters and the lack of evidence of overdispersion from clustering, the within-cluster correction was not included in the analysis. We have modified the text to clarify this: (Statistical Analyses under the Method section, page 6):

“ To address any overdispersion by clustering we considered a random effects model and a quasibinomial model but the appropriateness of the random effects model was limited by the small cluster sizes and small number of clusters^2^ and the quasibinomial model dispersion parameter was 1.0, indicating little evidence of overdispersion”

3. **Further discussion of the ELISA test performance would be useful – has the test been sufficiently evaluated?**

Thank you for the suggestion, we have added the following text to the manuscript under the method and limitation section as well as more references for test performance evaluation:

Method section (page 6)

“Dengue seropositivity was defined as the detection of immunoglobulin G (IgG) antibodies to dengue antigen serotypes (1, 2, 3, and 4) using the Panbio Dengue IgG indirect ELISA (enzyme-linked immunosorbent assays), a commercial capture ELISA with high specificity and sensitivity, and excellent correlation with Hemagglutination Inhibition (HAI) assay.^23-25^”

Limitation section (page 16)

“While the diagnostic test Panbio Dengue IgG Indirect ELISA is proven to be highly reliable and showed high agreement with HAI,^23^ there is a chance that a few study participants were misclassified in terms of seropositivity (false negative) with 97.9% sensitivity and 100% specificity.^22,71^”

**Reviewer #4:**

1. **The study is a baseline survey of cRCT, which supposed to be conducted in 68 clusters that were selected from 3,020 clusters in Fortaleza, Brazil. However, authors only managed to collect data from four clusters and partial data from 8 clusters. Are the 4 clusters representative for 3020 clusters?**

The study population is representative of the Fortaleza population when compared to the 2010 census. Please refer to Table 1 and the limitation section for more information.

2. **Authors mentioned that they collected partial data from eight clusters, what do they mean by partial data?**

We have clarified this in the manuscript but generally, we meant that we began data collection in these 8 clusters and were not able to complete the collection due to the field conditions as previously mentioned. We had a target sample size for each cluster (n=86) and therefore, we were not able to obtain this target but we did collect complete data (questionnaire and DBS) on all of the included participants within these clusters. We have clarified this in the manuscript:

Method Section (page 4)

“The team was able to obtain the target sample size (n=86 per cluster) in four clusters although there were eight clusters where this was not possible due to the field conditions during the time period of November 2019 and February 2020. In the incomplete clusters, complete data was collected (e.g., questionnaire and DBS) on a smaller number of participants within these clusters.

3. **A total of 392 households and 483 participant children were enrolled in the study. The sample size may be reasonable if authors used simple random sampling, but the sampling method was cluster sampling, which requires higher sample size.**

Thank you for the comment and this was a concern that was mentioned in the previous review. The 10x larger sample size was calculated for the primary objective of the cluster randomized trial (cRCT) and prior to data collection. For the primary objective of the cRCT, power calculations were based on the minimum clinically meaningful difference in prevalence of 0.2115 in those with the intervention and 0.30 in those without the intervention. The study in Fortaleza is terminated, so data collection has ceased, and we are restricted to these (partial) baseline data. The current KAP study objective is part of a secondary objective. In this post-hoc analysis, we are comparing seroprevalence between low, medium, and high KAP score categories, we are using the data available, and we are basing our understanding of any power limitations on the standard errors and width of the confidence intervals.

Nevertheless, we have performed retrospective power calculations based on the seroprevalences observed for each KAP category and, separately on the minimum clinically meaningful prevalence stated in the original intervention study protocol i.e. 0.2115 vs 0.3.

Calculation:

The power calculation inputs are the seroprevalence of each KAP category pair, the ICC, the coefficient of variation for the cluster sizes, and the average cluster sample size.

Seroprevalences by KAP category are 0.21, 0.26 and 0.39 for the low, medium and high KAP categories, respectively.

We have an ICC (Lohr rho) of 0.03

The coefficient of variation is (SD of cluster sizes divided by the mean), 37.7/40.25 = 0.94

With 12 clusters, a coefficient of variation of 0.94, a significance level of 0.05, and an average of 40 subjects per cluster, we would have 91 % power to detect a difference between the High and Low KAP group seroprevalences of 0.39 and 0.21, respectively. We have only 16% power to detect a difference of 0.26 and 0.21 prevalence between the medium and low KAP groups.

The original cluster randomized clinical trial considered differences based on seroprevalences of 0.21 (intervention group) and 0.30 (control group). The power to detect this difference is only 39%. However, as mentioned, this was not the primary objective of the current research study.

We also clarified this in the manuscript (Page 4 Method Section):

“The cRCT was powered for the primary objective of comparing seroprevalence between those receiving the intervention and those not receiving the intervention.^19^ The current study was part of a secondary objective, therefore a power analysis was not conducted. The cRCT planned to collect data from 68 clusters with a target recruitment of 5,848 children between 2 and 12 years old. For further detail, please see the original trial protocol in Zinszer *et al*. 2020.^19^

4. **It is not clear how did authors selected households in the selected clusters.**

All households that met eligibility criteria within the selected cluster were included, for more details, please refer to the following texts under Eligibility and Study Procedure section in the manuscript:

“Households in selected clusters were eligible to participate if there were children between 2 to 12 years old living in the household and if both the parent or legal guardian gave consent and the child gave assent for the data collection.”

5. **Are all children in the households were enrolled?**

Yes

6. **The presentation of results is poor:
   - Table 1 should be shortened
   - Table 2 is not well presented: I suggest one column for sample size N, one column for positive n(%), one column for 95%CI and one column for prevalence ratio.
   - Table 3 is not well presented, adding the descriptive data may make the table more informative
   - Do tables 4 – 6 add much?
   - Table 7: what about adding knowledge attitudes and practices presented in table 3 to the risk factors and use both Bivariate and multivariable analysis. In addition, the descriptive data makes the table more informative.**

Thank you for your suggestions and our tables were modified from our originally-submitted tables based on previous comments. We have made the following changes to the tables based on your feedback:

**Table 1:** We removed the household and children's sample size per borough and moved this information to supporting files.

**Table 2**: We modified the table based on your suggestion accordingly.

**Table 3:** We appreciate your suggestion. Table 3 displays the associations between each domain's score, as well as the associations between household serology results and KAP scores using data from all households. The focus of this table is on descriptive results that provide a general idea on the potential relationship between these variables we are interested in, rather than predictive outcomes like Table 7, therefore, we find it adequately fulfills its purpose.

**Table 4-6:** We moved these tables to supporting files.

**Table 7(now Table 4):** Thank you for your suggestion regarding Table 7. However, as one of the study’s objectives is to examine the predictors of seropositivity while controlling for confounders, we will not include the crude (bivariate) results.
